# Multifetal gestations after traumatic brain injury: a nationwide register-based cohort study in Finland

**DOI:** 10.1186/s12884-023-05539-z

**Published:** 2023-04-04

**Authors:** Matias Vaajala, Rasmus Liukkonen, Ilari Kuitunen, Ville Ponkilainen, Maiju Kekki, Ville M. Mattila

**Affiliations:** 1 Faculty of Medicine and Life Sciences, University of Tampere, Tampere, Finland; 2 Department of Pediatrics, Mikkeli Central Hospital, Mikkeli, Finland; 3 Institute of Clinical Medicine and Department of Pediatrics, University of Eastern Finland, Kuopio, Finland; 4 Department of Surgery, Central Finland Central Hospital Nova, Jyväskylä, Finland; 5 Department of Obstetrics and Gynecology, Tampere University Hospital, Tampere, Finland; 6 Center for Child, Adolescent and Maternal Health Research, Faculty of Medicine and Health Technology, Tampere University, Tampere, Finland; 7 Department of Orthopaedics and Traumatology, Tampere University Hospital Tampere, Tampere, Finland

**Keywords:** Multifetal gestations, Traumatic brain injury, Epidemiology, Pregnancy

## Abstract

**Background:**

There is a paucity of information regarding the association between traumatic brain injuries (TBIs) and subsequent multifetal gestations. Since TBIs are known to negatively affect the neuroendocrine system, we hypothesized that the functions of the whole reproductive system might be disturbed as a result. The aim of this study is to determine the association between previous TBIs and the risk of multifetal gestations using nationwide registers.

**Methods:**

In this retrospective register-based cohort study, data from the National Medical Birth Register (MBR) were combined with data from the Care Register for Health Care. All fertile-aged women (15–49 years) who had sustained a TBI before pregnancy were included in the patient group. Women with prior fractures of the upper extremity, pelvis, and lower extremity were included in the control group. A logistic regression model was used to assess the risk for multifetal gestation after TBI. Odds ratios (ORs) and adjusted odds ratios (aOR) with 95% confidence intervals (CIs) between the groups were compared. The model was adjusted by maternal age and maternal BMI during pregnancy and previous births. The risk for multifetal gestations were evaluated during different periods following the injury (0–3 years, 3–6 years, 6–9 years, and 9 + years).

**Results:**

A total of 14 153 pregnancies occurred after the mother had sustained a TBI, and 23 216 pregnancies occurred after the mother had sustained fractures of the upper extremity, pelvis, or lower extremity. Of these, 201 (1.4%) women had multifetal gestations after TBI and 331 (1.4%) women had multifetal gestations after fractures of the upper extremity, pelvis, or lower extremity. Interestingly, the total odds of multifetal gestations were not higher after TBI when compared to fractures of the upper extremity, pelvis, and lower extremity (aOR 1.04, CI 0.86–1.24). The odds were highest at 6–9 years (aOR 1.54, 1.03–2.29) and lowest at 0–3 years (aOR 0.84, CI 0.59–1.18).

**Conclusion:**

The risk for multifetal gestations after TBIs was not higher than after the other traumas included in this study. Our results provide good baseline information on the effects of TBIs on the risk for multifetal gestations, but further research is required on this topic.

## Background

There is a lack of information in the literature on the association between traumatic brain injuries (TBIs) and subsequent multifetal gestations. As both are relatively rare events, it is a truly challenging task to investigate them without the proper data. A previous Finnish nationwide study on birth rates after different traumas found that women with pelvic or hip fractures had lower birth rates than women in the general population. However, those women who had sustained a TBI had an even higher birth rate than the control group [1]. To our knowledge, this is the first time the effects of TBI on the risk for having multifetal gestations has been studied. The direct effects of TBIs on the neuroendocrine system, especially on the hypothalamic–pituitary complex, are well studied [2]. It is known, for example, that this structure, and therefore the whole hormonal system, including the targets of gonadal hormones, are vulnerable to damage and functional disturbances [2].

Since TBIs are known to negatively affect the neuroendocrine system [3, 4], we hypothesized that the functions of the whole reproductive system might be disturbed as a result [2, 5]. Our hypothesis is based on the findings of previous studies. TBIs are known to cause ovulatory disorders by affecting follicle stimulating hormone (FSH), which regulates the development of follicles, [6] and luteinizing hormone (LH), which is required for inducing ovulation. Thus, by causing ovulatory disorders, TBI could increase the risk for multifetal gestation [7]. Especially severe TBIs are also known to cause impairment and disruption of the hypothalamic–pituitary–gonadal axis [8–10]. Such injuries have been found to lead to GnRH dysregulation, altered LH, and FSH release, resulting in impairment of the production of estradiol [11] which, in turn, might possibly affect the regulation of the development of follicles and increase the risk for multifetal gestations. Furthermore, neuropathological processes, including damage to the pituitary–gonadal axis, can also affect reproductive outcomes even after a long period of time, [12] meaning that the effects of TBIs on the reproductive system are long lasting and should, therefore, be understood better. In addition, according to the results of a recent nationwide study in Finland, the incidence of TBI among fertile-aged women has increased strongly. Thus, further research on the effects of TBI on the later reproductive health of women is warranted [13].

This study aims to determine the association between previous TBI and the risk for multifetal gestations using nationwide registers.

## Methods

In this nationwide retrospective register-based cohort study, data from the National Medical Birth Register (MBR) were combined with data from the Care Register for Health Care. Both registers are maintained by the Finnish Institute for Health and Welfare. Data from the registers were then combined using the pseudonymized identification numbers of the mothers. Our data included fertile-aged women (15–49 years), and the study period was from 1 January 1998 to 31 December 2018. The study protocol was approved by the Finnish authority Findata. The requirement for informed consent was waived by Findata.

The coverage and quality of the Care Register for Health Care are good [14]. According to a validation study conducted in 2014, the Care Register for Health Care is excellent as determined by accuracy of diagnosis and by both the accuracy and coverage of procedural coding and external cause for injury [15]. All traumas sustained by women aged 15–49 years between 1998 and 2018 were included. International Classification of Diseases 10^th^ revision (ICD-10) codes were used to identify trauma patients. Women who had sustained a TBI before pregnancy were included in the patient group. Women with ICD-10 codes related to intracranial injury(S06.0-S06.9) or ICD-10 codes related to skull fracture(S02.0-S02.9) formed the patient group. Skull and facial fractures were included in the patient group, as they are known to usually be associated with at least mild intracerebral injuries [16, 17]. The control group was formed using the ICD-10 codes of upper extremity, pelvis, or lower extremity fractures. Upper extremity fractures included fractures of the shoulder and upper arm (S42.0-S42.9), fractures of the forearm (S52.0-S52.9), and fractures of the wrist (S62.0-SS62.4). Fractures of the pelvis included ICD-10 codes S32.1-S32.9, and lower extremity fractures included fractures of femur (S72.0-S72.9), fractures of knee and lower leg (S82.0-S82.9), and fractures of calcaneus or talus (S92.0-S92.1). As spinal cord injuries can negatively affect the outcome of a pregnancy and the functioning of the reproductive system and fertility, [18] fractures of the spine were excluded from the present study. Those women who sustained a TBI but also sustained a fracture on the control group list of fractures, were still located in the patient group, and were not allowed to occur in both groups.

**Fig. 1:**
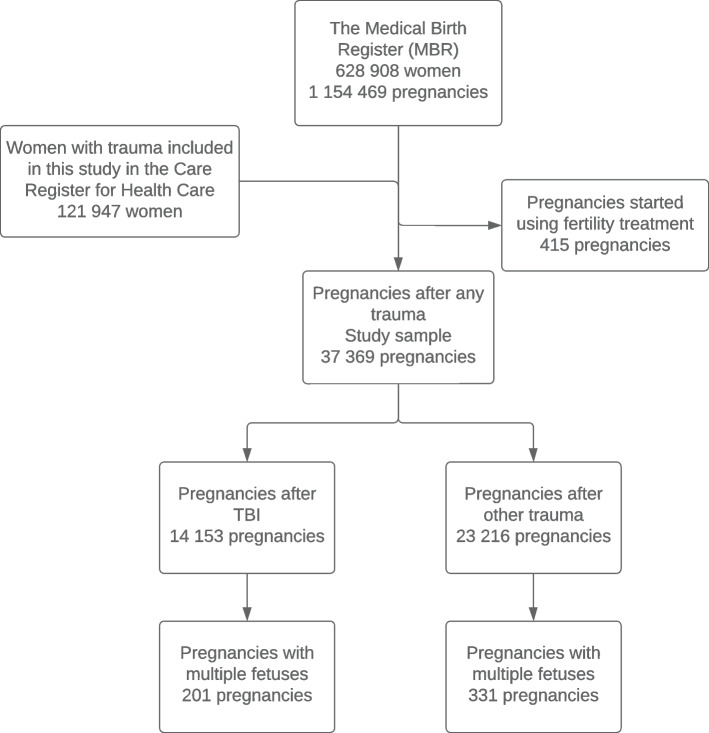
Flowchart of the study population. Pregnancies occurring after a TBI were compared to pregnancies occurring after fractures of the upper extremity, pelvis, or lower extremity

The MBR contains information on pregnancies, delivery statistics, and perinatal outcomes of all births with a birthweight of ≥ 500 g or a gestational age of ≥ 22^+0^. The MBR has high coverage and quality (the current coverage is nearly 100%) [19, 20]. We included all naturally conceived pregnancies that occurred between 1998 and 2018 after one of the traumas included in this study. Women who used fertility treatments were excluded from the analysis, as the chances of multifetal gestations are higher among these women [21]. The process used to form the study groups is shown as a flowchart in Fig. 1.

### Statistics

A logistic regression model was used to assess the primary outcomes. The exposure variable was the type of trauma, and the primary outcome was the risk of multifetal gestations. Odds ratios (ORs) and adjusted odds ratios (aORs) with 95% confidence intervals (CIs) between the groups were compared. The model was adjusted by maternal age and maternal body mass index (BMI) during pregnancy, as higher age and BMI are known to be risk factors for multifetal gestations [22, 23]. There is a paucity of information on the effects of TBIs on the subsequent risk for multifetal gestations, and the extent to which the adverse effects of TBIs affect the reproductive system remains unknown. Furthermore, as it is unclear whether the effects of TBIs occur with a delay, we also tested the risk for multifetal gestations in different time periods following the injury (0–3 years, 3–6 years, 6–9 years, and 9 + years). Statistical analysis was performed using R version 4.0.3 (R Foundation for Statistical Computing, Vienna, Austria).

### Ethics

Both the National Medical Birth Register (MBR) and the Care Register for Health Care had the same unique pseudonymized identification number for each patient. The pseudonymization was done by the Finnish data authority Findata. The authors did not have access to the pseudonymization key, as it is maintained by Findata. In accordance with Finnish regulations, no informed written consent was required because of the retrospective register-based study design and the patients were not contacted. Permission for the use of the data was granted by Findata after evaluation of the study protocol. (Permission number: THL/1756/14.02.00/2020).

## Results

A total of 14 153 pregnancies occurred after the mother sustained a TBI, and 23 216 pregnancies occurred after the mother sustained fractures of the upper extremity, pelvis, or lower extremity. Of these, 201 (1.4%) were multifetal gestations after TBI and 331 (1.4%) were multifetal gestations after fractures of the upper extremity, pelvis, or lower extremity. Maternal age during pregnancy was higher in the control group when compared to the TBI group (mean 30.4 years vs 28.6 years). Concussion (S06.0) was the most common type of TBI, occurring in approximately 87% of TBI patients. Diffuse TBI was another common type of TBI, occurring in 3% of patients. The absolute number of other TBI diagnoses was low. The majority of TBIs (84.1%) required less than one day of hospitalization, and only 1% of TBIs required a hospitalization period of more than 1 week (Table 1).

**Table 1:** Background information on the study groups

	TBI group		Control group	
Total number	14 153		23 216	
	n	%	n	%
Age at the time of trauma (mean; sd)	22.3 (5.5)		23.8 (5.9)	
Age at the time of pregnancy (mean; sd)	28.7 (5.5)		30.4 (5.4)	
Length of the hospitalization period (TBI only)				
< 1 day	11 908	84.1	-	
< 1 week	2104	14.9	-	
> 1 week	141	1.0	-	
Number of births before TBI				
nulliparous	6241	44.1	10 268	44.2
1	7653	54.1	7653	33.0
2	3270	23.1	3270	14.1
3 +	3230	22.8		
Total number of deliveries after trauma^a^				
1	8403	59.6	14 163	61.0
2	4928	34.8	6683	28.8
3 +	822	0.6	2370	10.2
Maternal smoking status during pregnancy				
Smoker	3954	27.9	4708	20.3
Unknown	420	3.0	679	3.0
Maternal body mass index (kg/m^2^) (mean; sd)	24.9 (5.2)		25.2 (5.3)	
BMI missing	1314	9.3	2761	11.9
Time difference between trauma and pregnancy				
0–3 years	4642	32.8	7091	19.5
3–6 years	3924	27.8	6328	26.9
6–9 years	2740	19.4	4696	20.0
9 + years	2975	21.0	5385	22.8
Multifetal gestations	201	1.4	331	1.4

^a^Only deliveries available in our data

The total odds for multifetal gestations were not higher after TBI when compared to other traumas (aOR 1.09 CI 0.90–1.31). The odds were highest at 6–9 years (aOR 1.50, 1.00–2.20) and lowest at 0–3 years (aOR 0.74, CI 0.48–1.10) (Table 2).

**Table 2:** Time stratified and overall time odds ratios (OR) and adjusted odds ratios (aOR) with 95% confidence intervals (CIs) for the event of having a multifetal gestation after a trauma

	OR (CI)	aOR (CI)
Total risk	1.00 (0.83 – 1.19)	1.09 (0.90 – 1.31)
Risk at 0–3 years after injury	0.78 (0.56 – 1.08)	0.74 (0.48 – 1.10)
Risk at 3–6 years after injury	0.87 (0.60 – 1.24)	1.09 (0.74 – 1.59)
Risk at 6–9 years after injury	1.43 (0.97 – 2.12)	1.50 (1.00 – 2.22)
Risk at 9 or more years after injury	1.14 (0.81 – 1.61)	1.16 (0.76 – 1.77)

Mothers who sustained traumatic brain injuries (TBIs) were compared to those who sustained fractures of the upper extremity, pelvis, and lower extremity. The model was adjusted with maternal age, maternal BMI, and previous births

## Discussion

The main finding of the present study is that it appears the odds of multifetal gestations after TBI were not higher when compared to the odds following fractures of the upper extremity, pelvis, and lower extremity. The odds for multifetal gestations 6–9 years after trauma were a little higher in the TBI group, but the clinical importance of this finding remains unclear. Adjusting the models with maternal age and maternal BMI during pregnancy and the number of previous births increased the odds for multifetal gestations, meaning that these factors most likely affect the risk for multifetal gestations, which is supported by the previous literature [22, 23]. However, as we are unaware of previous studies that have examined the effects of TBI on the risk for multifetal gestations, the results of this study provide baseline information on this subject. However, due to the rarity of multifetal gestations, further research on this topic using larger datasets is required.

Interestingly, the latest nationwide study on birth rates after traumas found that women with TBIs had a relatively high birth rate [1]. Even though TBIs are known to negatively affect the hypothalamic–pituitary complex and the endocrine system, especially the functions of gonadal hormones, [3, 4] it appears that alterations to the hormonal system do not increase the risk for multifetal gestations. However, based on the length of the hospitalization period, many patients required less than one day of hospitalization, suggesting that the majority of these traumas should be considered mild TBIs. Also, as most of the TBIs were concussions, indicating milder TBI, these results cannot be directly compared to severe TBIs, and further research is required on the topic. The results of this study should, however, provide useful general information about the effects of TBIs on reproductive health and should serve as the base of future research on this topic.

The main strength of our study is the use of large nationwide register data with excellent coverage and quality [19, 20]. The register data used in our study are routinely collected using structured forms with national instructions, which ensures good coverage and reduces possible reporting and selection biases. In addition, confounding was taken into account in this study, as the control group comprised women with other traumas whose background was presumably more like that of women with TBI than of women in the general population. In addition, other known confounding risk factors, such as maternal age, and maternal BMI, which are known to increase the risk for multifetal gestations, were also considered.

The main limitation of our study is the missing clinical information on the traumas included in this study (e.g., radiological findings, trauma mechanisms, and cause of fracture). Therefore, the severity of the TBIs is based solely on the length of the hospitalization period. In addition, traumas occurring before the age of 15 were not available in our data. Another limitation is that spontaneous or induced abortions remain unknown in our data. Therefore, those women who became pregnant but had a pregnancy loss cannot be identified. However, in our study miscarriages are affecting both study cohorts, which is why this is not a major limitation. To the best of our knowledge, there are no previous studies assessing the effects of previous TBIs on the risk for miscarriages, and this topic should be further researched. However, as long as the effects of TBIs and other traumas for the risk for miscarriages remain unknown, this should be noted in this study. In addition, women treated in the primary health care sector only are not available in our data, meaning that some of the mildest TBIs are not available.

## Conclusion

Based on our results, we found that the risk for multifetal gestations after TBIs was not higher than after the other traumas included in this study. Our results provide good baseline information on the effects of TBIs on the risk for multifetal gestations, but further research on this topic is required.

## Data Availability

The data that support the findings of this study are available from Findata, but restrictions apply to the availability of the data, which were used under license for the current study, and so are not publicly available. Data are, however, available from the Corresponding Author (e-mail: matias.vaajala@tuni.fi) upon reasonable request and with the permission of Findata (url Findata.fi, email info@Findata.fi).

## Abbreviations

TBI: Traumatic brain injury
OR: Odds ratio
CI: Confidence interval
